# Navigating Monogamy: Nonapeptide Sensitivity in a Memory Neural Circuit May Shape Social Behavior and Mating Decisions

**DOI:** 10.3389/fnins.2017.00397

**Published:** 2017-07-11

**Authors:** Alexander G. Ophir

**Affiliations:** Department of Psychology, Cornell University Ithaca, NY, United States

**Keywords:** hippocampus, lateral septum, mating system, memory, nucleus accumbens, prairie voles (*Microtus ochrogaster*), social behavior, retrosplenial cortex

## Abstract

The role of memory in mating systems is often neglected despite the fact that most mating systems are defined in part by how animals use space. Monogamy, for example, is usually characterized by affiliative (e.g., pairbonding) and defensive (e.g., mate guarding) behaviors, but a high degree of spatial overlap in home range use is the easiest defining feature of monogamous animals in the wild. The nonapeptides vasopressin and oxytocin have been the focus of much attention for their importance in modulating social behavior, however this work has largely overshadowed their roles in learning and memory. To date, the understanding of memory systems and mechanisms governing social behavior have progressed relatively independently. Bridging these two areas will provide a deeper appreciation for understanding behavior, and in particular the mechanisms that mediate reproductive decision-making. Here, I argue that the ability to mate effectively as monogamous individuals is linked to the ability to track conspecifics in space. I discuss the connectivity across some well-known social and spatial memory nuclei, and propose that the nonapeptide receptors within these structures form a putative “socio-spatial memory neural circuit.” This purported circuit may function to integrate social and spatial information to shape mating decisions in a context-dependent fashion. The lateral septum and/or the nucleus accumbens, and neuromodulation therein, may act as an intermediary to relate socio-spatial information with social behavior. Identifying mechanisms responsible for relating information about the social world with mechanisms mediating mating tactics is crucial to fully appreciate the suite of factors driving reproductive decisions and social decision-making.

## Introduction

The nonapeptides vasopressin (VP) and oxytocin (OT), and their non-mammalian homologs, are crucial regulators of social behavior across taxa (Goodson and Bass, 2001; Goodson, 2008; Goodson and Thompson, 2010). Indeed they are well known for their roles in social behavior, pairbonding, and mating systems (Young and Wang, 2004; Carter et al., 2008; Insel, 2010), including in humans (Heinrichs et al., 2009). It is less recognized that they were first studied in behavioral neuroscience for their central effects on memory (de Wied, 1971; Bohus et al., 1978b; Pedersen and Prange, 1979; McEwen, 2004). Somewhat ironically, mating systems are inherently dependent on social and spatial memory. For example, most theories of the evolution of mating systems emphasize the importance of space use.

Unfortunately, how animals use information about the spatial distribution of conspecifics and the social context of interactions to inform mating decisions has been drastically underappreciated. Individual mating tactics should reflect the social landscape (i.e., context) in which animals find themselves. For example, most males must consider the defendable resources located within a given territory, the number of mating partners they are capable of monopolizing, and the activity of their mate(s) and neighbors (Emlen and Oring, 1977; Shuster and Wade, 2003). Based on their best estimate of the context and the status of their own body condition, individuals should adopt a mating tactic that will most likely produce the greatest reproductive success. The assessment of these factors will largely depend on integrating social and spatial information (i.e., the identity and location of potential mates or competitors). This process requires behavioral coordination by multiple neural mechanisms, including a major role for the action of VP and OT in the forebrain.

Here, I begin by discussing theory for mating systems emphasizing space use as a driving force. I then introduce and briefly review the nonapeptide neuromodulatory system (vasopressin and oxytocin) and its role in pairbonding and memory. I next outline the connectivity between several neural structures in which nonapeptides assert an influence and may serve as the foundation for a putative “socio-spatial memory neural circuit”. Finally, I speculate on how this putative circuit may function and interact with other known circuits to influence reproductive decisions and mating tactics using prairie voles as a case study example. Although, some attention has been dedicated to understanding female prairie vole reproductive decision-making and the mechanisms therein (Zheng et al., 2013b) in this review I primarily consider males, unless otherwise stated. While many of the theoretical and presumably neuroanatomical details are likely to generalize between the sexes, sexual selection and sex-dependant selection have also produced important differences that are likely to alter the proximate and ultimate processes that shape reproductive decision-making in males and females. It is crucial to consider both sexes if we are to ever achieve a full understanding of the mechanisms that contribute to such important decisions, and the dynamic interactions that follow. Nevertheless, deepening our concept of mating systems by incorporating memory represents significant progress toward understanding how neural mechanisms govern complex behavior.

## How might social or spatial memory influence mating systems?

Ultimately, mating systems are social systems that represent the outcomes of several individual reproductive choices. Indeed, mating systems are emergent properties of populations, whereby each individual assesses the ecological and social landscape in which they find themself and adopts a tactic based on that information (Oliveira et al., 2008). In this review I use terms like “assess”, “adopt”, and “evaluate” in the behavioral ecology sense of the words. This is meant specifically to reflect information gathering (however reliable or unreliable) and the outcome of a computation in which that information was used to inform the probability that the world is in a particular state, and weighed against the expected gains of performing various behaviors given that state. From this perspective, “cognition” is deeply rooted in the behavioral ecology of all species (Sherry, 2006; Dukas and Ratcliffe, 2009). Identifying the social and cognitive factors that sculpt mating decisions is necessary to predict reproductive success, and to understand how individual decisions contribute to and shape social organization at large.

At their core, mating systems represent the most common social arrangements of individuals of a given population or species. They are often synonymously conflated with breeding arrangements and portrayed as a collection of mating decisions for the purpose of reproduction. It should be stated that how animals mate and how they live may not perfectly map on to each other, and breeding arrangements and social arrangements often differ (Kleiman, 1977). Indeed, alternative tactics commonly evolve in mating systems, and frequently take the form of territorial vs. “sneaker” tactics, although other forms of alternative reproductive tactics also exist (Oliveira et al., 2008).

In simple terms, mating systems can be characterized by the average number of mates (or at least social partners) that males and females of a given population most commonly have (Shuster and Wade, 2003). This way of categorizing reproductive behavior creates four general categories of mating systems: polygyny (one-male—multi-female units), polyandry (one-female—multi-male units), promiscuity (a.k.a., polygamy, polygynandry; multi-male—multi-female units), and monogamy (one-male—one-female units). Just how common each mating tactic is varies by taxa. For example, although monogamy is common in birds (Owens and Bennett, 1997), it is very rare among mammals (Kleiman, 1977). Nevertheless, monogamy is particularly interesting considering that humans represent one mammalian species that readily engages in this mating system. For reviews of mating systems see Apollonio et al. (2000); Clutton-Brock (1989); Greenwood (1980); Orians (1969); Shuster and Wade (2003).

Although, mating systems are conceptualized in terms of reproductive behaviors, they are frequently defined in terms of social and spatial behavior. Emlen and Oring (1977) argue that the potential for polygyny is contingent on a male's ability to monopolize resources that attract females by establishing large territories (an entity inherently rooted in space). In fact, the size and exclusivity of territories are measures commonly used to define mating systems. For example, mammalian monogamy is most likely to evolve when females occupy small but exclusive home ranges, thereby increasing the difficulty for males to monopolize several females (Komers and Brotherton, 1997).

In practice, a male must be proficient at defending resources while monitoring the activity of his mate or mates to successfully mate-guard or exclude competitors. Presumably, polygynous (and promiscuous) males must track the temporal progression of the estrous cycle of nearby females, establish territories, and remember neighbors (Brotherton and Komers, 2003). Males that are in the right place at the right time are likely to experience a reproductive advantage over others. Alternatively, a monogamous male must monitor the activity of his neighbors, both in terms of which individuals directly threaten his fitness through cuckoldry or infanticide, and indirectly through challenging his resource holding potential (Brotherton and Komers, 2003). He may also benefit greatly by tracking the identity and location of neighbors that may boost his fitness through pairing (or extra pair) opportunities (Brotherton and Komers, 2003). Monogamy should therefore require males to associate the spatial distribution of conspecifics with their social identity: are they competitors, pairbonded mating partners or potential extra-pair mates? Ultimately, a male's decision to adopt a particular mating tactic should be informed by associations with space use (*spatial memory*), distinguishing between neighbors (*social memory*), and accounting for the spatial distribution of those neighbors (*socio-spatial memory*).

## The socially monogamous prairie vole

Perhaps no species is better studied with resect to both natural behavior comprising their mating system and the neurobiological mechanisms that govern important aspects of the mating system than prairie voles (*Microtus ochrogaster*). Prairie voles overwhelmingly demonstrate behaviors consistent with a monogamous mating system (e.g., Thomas and Birney, 1979; Gavish et al., 1981; Getz et al., 1981; Carter and Getz, 1993). In the lab, males and females form long-lasting social preferences for a mating partner (i.e., a pairbond), demonstrate aggressive behavior toward strangers, and provide care for offspring (Witt et al., 1988; Williams et al., 1992; Winslow et al., 1993; Carter et al., 1995). In the field, prairie voles tend to form breeding units in which one male and female breeder attend to offspring until they disperse (McGuire et al., 2013). Animals adopting this mating tactic are often referred to as “residents”, largely because males and females occupy overlapping home ranges and appear to exclude other conspecifics (Getz et al., 1993; Solomon and Jacquot, 2002; Ophir et al., 2008a).

The majority of males adopt residency (~60–75%), one reason why prairie voles are considered monogamous (Getz et al., 1993). But being a monogamous species need not exclude males or females from attempting to engage in extra-pair mating. In fact, resident males face a trade-off between mate guarding to increase “faithful” in-pair reproduction and “unfaithful” extra-pair mate seeking (Ophir et al., 2008a; Phelps and Ophir, 2009; Okhovat et al., 2015). Here, I refer to the former as “*true residents*” and the latter as “*roving residents*” or simply “*rovers*”. The proportion of these two types of residents has not been formally investigated, but some studies hint that they are roughly equally common (Getz et al., 1993; Ophir et al., 2008a; McGuire and Getz, 2010; Okhovat et al., 2015). Home range size of true residents and rovers are similar, but the way they use the same amount of space might differ. For instance, how rovers and true residents navigate and move within the same area and interact with conspecifics is almost surely different and would reflect their chosen tactics. This would most likely express itself in the degree to which they remain at the nest and mate guard and the degree to which they visit the nests of other females.

Beyond true residents and rovers, another important and less common (~25–30% of the population) tactic referred to as “*wandering*” exists. In this case, male and female wanderers occupy much larger home ranges compared to residents, they do not appear to be territorial, and they presumably attempt to mate multiply (Getz et al., 1993; Solomon and Jacquot, 2002; Ophir et al., 2008a; McGuire and Getz, 2010; McGuire et al., 2013). It is unclear if adopting a resident or wandering tactic produces greater reproductive payoffs, but our data suggest that males prefer to form bonds (Blocker and Ophir, 2016) and that residents may have greater reproductive success while wanderers are making the best of a bad job (Ophir et al., 2008a; Phelps and Ophir, 2009). Taken together, it is most accurate to characterize the overall mating system of prairie voles as socially monogamous, in which animals engage in alternative reproductive tactics.

## Nonapeptides, monogamy, and memory

Oxytocin and vasopressin are integral to many forms of mammalian social behavior (Goodson, 2008; Goodson and Thompson, 2010). Although knowledge of the function of nonapeptides in social behavior across an array of species has progressed at a remarkable pace, a foundation of knowledge has been built around prairie vole social behavior, affiliation, and pairbonding. Indeed, nonapeptides are necessary and sufficient for the production of prairie vole pairbonds (Young and Wang, 2004; Johnson and Young, 2015)—a hallmark of their socially monogamous mating system. For example, manipulation of VP and its receptor (V1aR) in the ventral pallidum (VPall) or lateral septum (LS), and OT or its receptor (OTR) in the nucleus accumbens (NAcc) can either facilitate or diminish the pairbond (Winslow et al., 1993; Williams et al., 1994; Cho et al., 1999; Liu et al., 2001; Liu and Wang, 2003; Lim et al., 2004; Ross et al., 2009; Keebaugh et al., 2015). These and other limbic structures, sometimes referred to as a “*pairbonding neural circuit*” (Young and Wang, 2004), have formed the basis for understanding the neurobiology of social affiliation and monogamy (for reviews see Carter and Keverne, 2002; Young and Wang, 2004; Carter et al., 2008; Donaldson and Young, 2008; Insel, 2010; McGraw and Young, 2010; Carter, 2014; Lieberwirth and Wang, 2014; Johnson and Young, 2015).

The expression of nonapeptide receptors across the brain can reveal how evolution has shaped the mechanisms that impact mating decisions (Ketterson and Nolan, 1992). For example, studies famously comparing monogamous and non-monogamous vole species indicate that nonapeptide receptor profiles (particularly within the aforementioned areas) are good predictors of mating system (Insel and Shapiro, 1992; Insel et al., 1994). Similar characterizations have since been performed in many other species with different mating systems or social organization (c.f., Kelly and Ophir, 2015). How broadly the relationship between nonapeptides and mating system extends beyond voles is unclear, but some evidence suggests parallel results may exist for humans and chimpanzees (Hammock and Young, 2006; Donaldson et al., 2008; Walum et al., 2008; Hopkins et al., 2012). Strangely, the extraordinary individual variation in prairie vole V1aR or OTR density does not differ between residents and wanderers (Ophir et al., 2008b, 2012; Zheng et al., 2013b). Evidence demonstrating that residents produce more fertilized embryos (Ophir et al., 2008a) suggests that natural selection has eliminated the standing variation in the pairbonding neural circuit to predispose prairie voles to adopt a socially monogamous lifestyle. It should be noted that this conclusion is built on the assumption that unborn embryos are a rough proxy of fitness. However, this measure does not account for variation in parental care these offspring would have received, the lifetime reproductive success of the breeding unit, or ultimately survival and subsequent reproduction of the offspring (see Ophir et al., 2008a; Blocker and Ophir, 2016) which might have altered the “fitness advantage” of bonded males in either direction. Nevertheless, if there is indeed a reproductive advantage to being paired, then the mechanisms that promote pairing should be advantageous to all males. Therefore, any individual variation in the neural phenotype that is known to facilitate (or gate) bonding, should be low and all males have the same “bonding” neural phenotype, more or less. Our neural data appear to support this interpretation.

Considerable evidence from the mid-twentieth century demonstrated that OT and VP affect the process of learning and memory, either directly or indirectly by altering arousal (c.f., McEwen, 2004). The original work in this area focused on the impact of VP and OT in passive or active avoidance learning (Bohus et al., 1972, 1978b; de Wied, 1991), but has also expanded to understanding navigation (i.e., hippocampal-dependent cognition; e.g., Engelmann et al., 1992; Everts and Koolhaas, 1999), retrieval and relearning in visual discrimination (Alescio-Lautier et al., 1987), and social recognition and social memory (e.g., Ferguson et al., 2002; Albers, 2012; Stevenson and Caldwell, 2012). The main neural targets on which VP and OT assert effects on memory include the hippocampus, the cingulate and retrosplenial cortices, septum, several subunits of the thalamus, hypothalamus, and other limbic structures such as the amygdala and medial preoptic area (e.g., Popik and Van Ree, 1998; Ferguson et al., 2001; McEwen, 2004; Ophir et al., 2008b). More recently, increasing attention has been dedicated to understanding the roles of nonapeptides in the hippocampus and hippocampal-dependent memory. For instance, Egashira et al. (2004) showed that vasopressin is necessary to perform a hippocampal-dependent spatial memory task, and Tomizawa et al. (2003) showed that hippocampal oxytocin may be necessary for long-lasting spatial memory. Interestingly, OT appears to enhance hippocampus spike transmission by modulating fast-spiking interneurons, effectively improving the signal-to noise ratio (Owen et al., 2013).

Much of the evidence has led to the idea that VP and OT appear to have opposite effects on learning and memory, with VP facilitating memory consolidation and retrieval, and OT potentially serving as an amnestic (Bohus et al., 1978a; Kovacs and Telegdy, 1982; Argiolas and Gessa, 1991; de Wied, 1991; McEwen, 2004). For example, nonapeptides are functionally important for social recognition (Gabor et al., 2012). Blockade of endogenous VP in the septum and the hippocampus (dorsal and ventral portions) disrupts social recognition, whereas OT blockade only impacts social recognition when administered to the ventral hippocampus and not the septum or dorsal hippocampus (van Wimersma Greidanus and Maigret, 1996). More specifically, central and peripheral injections of VP facilitate social recognition, whereas OT injections have no effect or attenuate it (Bohus et al., 1978a,b; Koob et al., 1981; Dantzer et al., 1987; Popik and Vetulani, 1991; Benelli et al., 1995). The role of OT on memory is much less clear than that of VP. In fact OT appears to have a dose-dependent effect on memory. High doses of OT produce amnestic effects but low doses facilitate recognition (Popik et al., 1992a,b). OT's dose-dependent influence on social recognition is probably explained by the types of OT metabolites that bind to OTR (Burbach et al., 1983; Popik et al., 1996; Popik and Van Ree, 1998), but could also be explained by OT-V1aR cross reactivity (de Wied, 1991; Manning et al., 2008; Song et al., 2014, 2016). Moreover, differences in how and where VP and OT impact social recognition appear to vary by sex (Gabor et al., 2012). Nevertheless, V1aR antagonists clearly block social recognition (Engelmann and Landgraf, 1994; Landgraf et al., 1995), while OTR antagonists block the facilitating effects of OT on social recognition at low doses and the attenuating effects of social recognition at high doses (Benelli et al., 1995).

As a cautionary warning, these results, particularly those regarding OT just discussed, highlight the importance of considering the route of administration, dose, timing, and the behavioral tests that are used to assess learning and memory. Administration and dose matter because both can potentially have confounding effects of arousal (Baldi and Bucherelli, 2005). Furthermore, nonapeptides do not readily cross the blood-brain barrier and therefore peripheral (e.g., intranasal or intraperitoneal injections) and central (e.g., targeted or intracerebroventricular) administration can have different results (Neumann et al., 2013). It is also unclear if peripheral administration has direct or indirect effects. Moreover, the administration of exogenous nonapeptides may have important and different dose-responses, as conveyed in the example given above. The timing of administration of pharmacological agents, or the like, may also impact memory in different and important ways because they may impact acquisition, consolidation, and/or expression of memory, which each follow different timelines. Finally, the behavioral test matters because the behavior of interest will change in line with the expected procedure for most behavioral tests of memory, however “memory” is an interpretation of the observed behavior (e.g., olfactory inspection, or visits to a particular area in space) rather than an observable behavior itself. Factors such as these are important to be mindful of when interpreting the studies that have investigated learning and memory and in particular the influences of nonapeptides on these processes, and may help explain why results may appear contradictory (e.g., why OT might appear to both facilitate and attenuate memory, see above).

## A functional memory neural circuit

A tremendous effort has been dedicated to describing and understanding the processes of learning and memory and the neural mechanisms that govern these processes. It is not my intent to review this entire literature here. However, I do aim to provide a brief and somewhat simplified synopsis of the neural circuit and structures therein that are closely associated with memory. For more exhaustive reviews of this topic see Aggleton and Brown (1999); Brown and Aggleton (2001); Eichenbaum et al. (2007); Fanselow and Dong (2010); Strange et al. (2014), and Zola-Morgan and Squire (1993).

### The hippocampus

The hippocampus (HPC) is probably the best-known neural structure associated with learning and memory. It is necessary for many forms of higher-level memory including episodic memory (i.e., recalling experienced events), contextual memory, and spatial memory (Hirsh, 1974; Nadel et al., 1985; Zola-Morgan and Squire, 1993; Rolls, 1996; Mizumori, 2007; Smith and Bulkin, 2014; Strange et al., 2014; Bulkin et al., 2016). Indeed some have characterized the HPC as a structure that generates representations of multi-dimensional spatial maps, while others have argued it is a center for assessing context (Hirsh, 1974; Nadel et al., 1985; Zola-Morgan and Squire, 1993; Rolls, 1996; Mizumori, 2007; Smith and Bulkin, 2014; Strange et al., 2014; Bulkin et al., 2016). The hippocampus is a highly conserved forebrain structure that takes its name from the curved sea horse-like shape it takes in the human brain. In rodents the HPC is shaped more like a cashew that curves at an angle along the anterior-posterior and dorsal-ventral axes. Although the orientation and location of the HPC has been rotated and drifted over mammalian evolution, this structure still maintains its basic configuration: the dorsal HPC (sometimes referred to as the septal pole, and represented by the anterior component of the HPC in rodents), and the ventral HPC (a.k.a., the temporal pole, representing the posterior component of the rodent HPC) (Strange et al., 2014). Although the cellular anatomy and connectivity within the HPC is fairly consistent throughout the length of the HPC, these two components appear to be functionally distinct, with the dorsal HPC accounting for the episodic memory, spatial map and navigational functions, and the ventral HPC relating to emotional memory, affect, and stress (Moser and Moser, 1998; Fanselow and Dong, 2010). Homologs of this structure take many forms in other taxa, like the HPC-equivalent found in the dorsal pallium in birds, or the aptly named mushroom bodies of some insects (Strausfeld et al., 1998; Kempermann, 2012). In mammals, the neuroanatomy and connectivity within the HPC is captured by the so-called “tri-synaptic loop”, a one-way loop of axonal connections from the entorhinal cortex (EC), penetrating through the subiculum, and through the sub-structures of the HPC (the dentate gyrus, CA1, and CA3) (Amaral and Witter, 1995; Brewer et al., 2013). To complete this loop, the axons of the cells in CA1 project to the neurons of the EC and subiculum.

**Figure 1:**
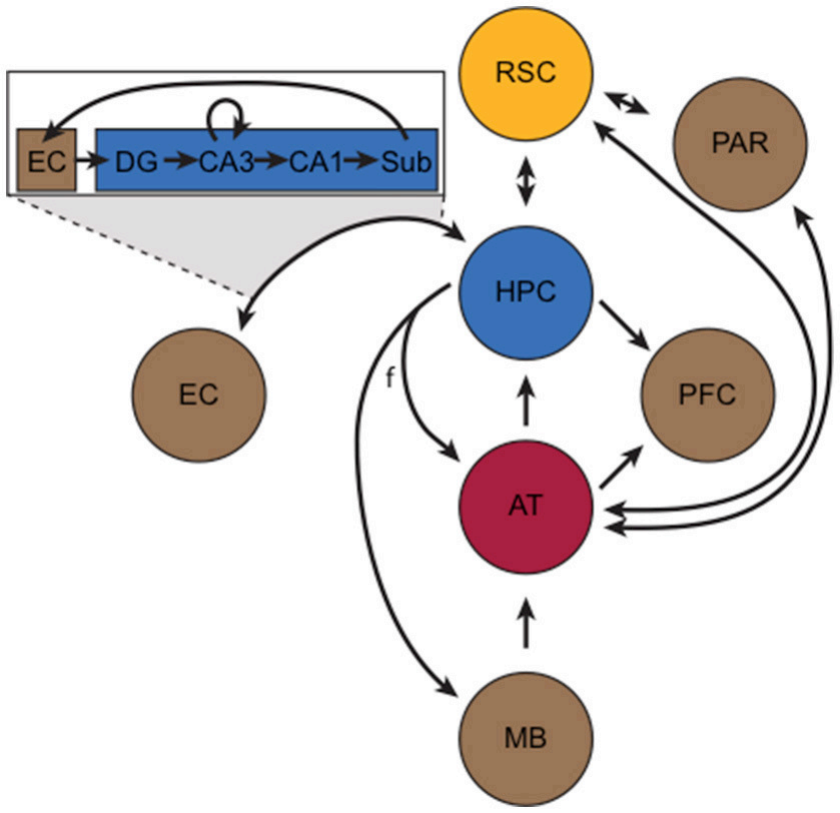
The hippocampus-anterior thalamic axis (adapted from Aggleton and Brown, 1999). At its core, the circuit incorporates the hippocampus (HPC, blue), retrosplenial cortex (RSC, yellow), and the anterior thalamus (AT, red). Other important structures (in brown), include the mammillary bodies (MB), entorhinal cortex (EC), parietal cortex (PAR), and the prefrontal cortex (PFC). The fornix (f) and other axonal projections are represented by black arrows. The box portrays the “tri-synaptic loop” between the EC and the subunits of the HPC, including the subiculum (Sub), dentate gyrus (DG), and CA1 and CA3 subfields.

Despite its central role in learning and memory, the HPC is functionally and anatomically connected with several other structures that work in concert to enable many important aspects of learning and memory. This extended memory circuitry has been described in detail elsewhere (Gabriel, 1993; Aggleton and Brown, 1999; Mizumori et al., 2000; Smith et al., 2012). Briefly, the so-called hippocampus-anterior thalamic axis (Figure 1) forms the basis of this circuit and incorporates the HPC, the fornix, mammillary bodies, retrosplenial cortex (RSC), and thalamic nuclei. The HPC sends and receives projections to/from the anterior thalamus (AT) via the fornix, and projects to the mammillary bodies via the fornix. The HPC, however, is also bidirectionally connected to the EC and RSC, and sends projections to the prefrontal cortex. Signals entering the HPC from the EC initiate the tri-synaptic loop. In addition to its bidirectional connection with the HPC, the RSC is bidirectionally connected with the parietal cortex and the AT.

### The retrosplenial cortex

The RSC is a key component of the brain's memory and navigation systems (Vann et al., 2009; Miller et al., 2014) and is located along the posterior portion of the cingulate cortex (Jones et al., 2005). Lesions to the RSC are remarkably similar to the effects of hippocampal lesions, including impairments in episodic memory (Valenstein et al., 1987; Bowers et al., 1988), contextual memory (Keene and Bucci, 2008a,b), and spatial navigation (Sutherland et al., 1988; Harker and Whishaw, 2002; Vann and Aggleton, 2002). Not only is the RSC bi-directionally connected with other components of itself (van Groen and Wyss, 1992a,b), but the HPC and RSC are reciprocally interconnected (Wyss and Van Groen, 1992; Jones and Witter, 2007), and this bidirectional communication is likely critical for memory. Inactivation of the RSC disrupts hippocampal representations (Cooper and Mizumori, 2001), and it appears to be an important consolidation target for hippocampal-dependent memories (Katche et al., 2013), especially contextual memories (Keene and Bucci, 2008c; Cowansage et al., 2014; Czajkowski et al., 2014).

### The anterior thalamus

The AT (consisting of the anterior dorsal, anterior ventral, anterior medial, and lateral dorsal nuclei) is bidirectionally connected with the HPC and RSC (synapsing in the granule layers of the RSC) (van Groen et al., 1993). The AT is a major subcortical target for HPC output (Swanson and Cowan, 1977; Aggleton et al., 1986), and like the RSC, it is involved in many of the same memory and navigation functions as the HPC. For instance, the AT is a critical site of damage in diencephalic amnesia (Aggleton et al., 2011). In rodents, AT neurons exhibit directional firing (i.e., head direction cells; Taube, 1995) and AT lesions reliably disrupt spatial navigation (Aggleton and Nelson, 2015), sequence memory (Wolff et al., 2006), and contextual memory (Law and Smith, 2012). Furthermore, AT lesions cause large-scale disruption of HPC and RSC functioning (Jenkins et al., 2002, 2004; Savage et al., 2011). In addition to the aforementioned connectivity, the AT also sends afferents to the prefrontal cortex and to the parietal cortex. In turn, the AT receives efferents from the HPC (via the fornix), mammillary bodies, and RSC. Similarly, the parietal cortex receives input from several structures, but most notably the dorsal RSC and laterodorsal (LDTh) subdivision of the AT. Moreover, the LDTh acts as a transitional nucleus projecting to both limbic and neocortical areas, and the presence of head direction cells in this structure is an interesting point of convergence with other areas central to spatial cognition (Mizumori and Williams, 1993; Taber et al., 2004). Anatomically, the LDTh provides extensive afferent input to the subicular complex of the hippocampal formation and sends dense projections to the RSC (van Groen and Wyss, 1992b). Indeed, the RSC is bi-directionally connected with the LDTh (van Groen and Wyss, 1992a,b). Taken together, the HPC, RSC and AT (including the LDTh) are central components of an extended limbic memory circuit that is vastly important for mediating spatial, episodic, and context dependent memory (Figure 1).

### Other structures: the septum and septohippocampal nucleus

It is important to make clear that the classic functional memory neural circuit just discussed is a relatively simplified model. Extending this model, the HPC, septohippocampal nucleus (SHi), and septum form a reciprocal circuit among themselves (Rye et al., 1984; Gaykema et al., 1990), which is directly involved in memory (Khakpai et al., 2013). Both the dorsal and ventral portions of the HPC project to the LS (Fanselow and Dong, 2010; Strange et al., 2014). Although the LS receives massive glutamatergic fiber input from the hippocampus via the fornix, the medial septum (MS) sends significant cholinergic and GABAergic projections to the hippocampus (Jakab and Leranth, 1995; Swanson and Risold, 2000). Meanwhile, the SHi, which is centrally involved but not necessary for learning and memory (Parent and Baxter, 2004), provides feedback between the HPC and (primarily) the MS (Giovannini et al., 1994; Marighetto et al., 1994). Interestingly, lesions of the medial septum disrupt hippocampal theta oscillations (Lawson and Bland, 1993) and impair spatial memory (Winson, 1978; Leurgeb and Mizumori, 1999). Thus, the septal connections back to the HPC and SHi primarily travel through the MS. However, the medial and lateral septa are themselves tightly and reciprocally connected to each other, and the LS can inhibit HPC function via the MS-SHi (Giovannini et al., 1994; Marighetto et al., 1994; Jaffard et al., 1996; Desmedt et al., 1999). Feedback through indirect LS regulation of the HPC via the SHi is accomplished through the glutamatergic receptors in the LS that exert an inhibitory effect of cholinergic cells in the MS, which in turn influences HPC function (Giovannini et al., 1994; Marighetto et al., 1994; Jaffard et al., 1996; Desmedt et al., 1999). Thus, there are cytoarchitectural and functional connections among the septum and hippocampus enabling direct communication from the HPC to LS and indirect LS feedback regulation of the HPC via the SHi.

## The social decision-making network and the pairbonding neural circuit

Central to the study of social behavior is a core set of interconnected limbic structures, collectively recognized as the social behavior network (SBN). These include the LS, preoptic area (POA), central and medial amygdala (CeA and MeA), bed nucleus of the stria terminalis (BST), anterior hypothalamus (AH), ventromedial hypothalamus (VMH), and midbrain (i.e., periaqueductal gray, PAG) (Newman, 1999; Goodson, 2005). By definition, these core nodes of the SBN are involved in the regulation of many forms of social behavior, are reciprocally connected, and are influenced by sex steroid hormones (Newman, 1999). For example, various combinations and permutations of the activation of these structures are necessary or important for the expression of sexual behavior, aggression, parental care, and social grouping within and across species (c.f., Numan, 2015). Somewhat recently, the SBN was extended to integrate reward circuitry into a larger network, called the social decision-making network (SDMN), comprised of the SBN structures and the NAcc, VPall, striatum, basolateral amygdala, ventral tegmental area (VTA), and notably the HPC (O'Connell and Hofmann, 2011a,b, 2012). These latter structures are tightly networked key nodes in or key accessories to the mesolimbic reward system (for review see Ikemoto, 2010). The mesolimbic reward system has become commonly regarded as the neural network where salience and valence of stimuli is processed (Alcaro et al., 2007; Wickens et al., 2007; Ikemoto, 2010). Dopamine, particularly in projections from the VTA to the NAcc, is a major factor in this function, but of course it is not the only important signaling molecule (Spanagel and Weiss, 1999). Much of the work on the mesolimbic reward system has been done under the premise of understanding mood disorders, addictive behavior, or reinforcement learning (Berridge and Robinson, 1998; Alcaro et al., 2007; Koob and Volkow, 2010; Dichter et al., 2012). But clearly natural, and in particular social, behavior heavily relies on reward (Schultz, 2000, 2006), which is probably one of the reasons this network appears to be ubiquitously shared across taxa. For an extensive review on the structure, function, and connectivity of nodes of the SDMN across four major vertebrate taxa, see (O'Connell and Hofmann, 2011b).

Although, no mention of nonapeptides was made in the original characterization of the SBN (Newman, 1999), these structures are largely sensitive to VP and OT action (Albers, 2015). For example, all of the SBN/SDMN structures, with the exception of the POA and VTA (but see Hammock and Young, 2005), express V1aR, OTR, or both in prairie voles (Zheng et al., 2013b). Not surprisingly, all nodes of the pairbonding neural circuit described above (see Young and Wang, 2004) are contained within the SDMN (with the one exception of the prefrontal cortex, which might also be important to include). Arguably, the decision to form a pairbond falls safely within social decision-making, and from this point of view it is reasonable to consider the pairbonding neural circuit as a sub-unit of the SDMN.

## A case for socio-spatial memory as a factor for mating system

The relationship between social and spatial memory, mating decisions, and the role of nonapeptides therein is likely to be much more than coincidental. Social decision-making necessarily relies on an individual's ability to assess the social and spatial landscape in which it finds itself and then act on that information. Such decision-making should be context dependent and plastic, yet open to the stabilizing or canonizing forces of natural selection. The action of nonapeptides as modulators of social behavior provides a plausible mechanism by which such plasticity can be maintained by natural selection. Indeed, individual differences in nonapeptide receptor expression may contribute to differences in socio-spatial memory and to differences in mating tactics, possibly as a consequence of its impact on memory. For instance, non-monogamous male meadow voles (*M. pennsylvanicus*) perform better than monogamous prairie voles in several mazes testing spatial memory (Gaulin and FitzGerald, 1989). Interpretations of these and other related results indicate that spatial memory (i) may facilitate navigating larger home ranges, (ii) differs systematically between mating systems, and (iii) potentially helps shape mating systems (Gaulin and FitzGerald, 1989; Jones et al., 2003), supporting the idea that memory is important for mating decisions. How social and spatial memory might operate within species to shape, and possibly promote, particular mating decisions is an open question and may vary based on the species under investigation.

Of particular importance here is the function of VP and OT in the RSC and HPC, respectively. Some polygamous rodents have larger HPC or RSC than monogamous congeners (Gaulin and FitzGerald, 1989; Gaulin, 1992; Clint et al., 2012; Jasarevic et al., 2012; Kingsbury et al., 2012), implicating these brain areas as being important for mating systems. Although interspecific comparisons of nonapeptide receptors in the HPC or RSC are limited, Insel et al. (1991) demonstrated that promiscuous mice (*Peromyscus maniculatus*) express more oxytocin receptor in the hippocampus (CA1 sub-region) than a monogamous congener (*P. californicus*), providing some of the first evidence that variation in nonapeptide receptor expression might relate to mating system.

In contrast, we have found no evidence suggesting that RSC or HPC *volume* predicts mating tactics within prairie voles (Kingsbury et al., 2012; Rice et al., in review). However, although size and volume of brain structures are commonly linked with information processing and its behavioral consequences (e.g., Sherry et al., 1992; Maguire et al., 2000), sheer size of structures is only one aspect of neural function. The neural mechanisms that operate within structures can also have a profound influence on neural processing and behavior (Roth et al., 2010). To this end, expression patterns of nonapeptide receptors within these structures predict successful adoption of monogamous or non-monogamous tactics (Ophir et al., 2008b, 2012; Okhovat et al., 2015, see below). This suggests that the most successful residents are more sensitive to VP and OT binding in these brain areas than the most successful wanderers. Overall, variation of VP and OT receptor expression within regions associated with memory processing appears to reflect the variance in the sensitivity to these neuromodulators, and hence their ability to impact memory, particularly for socially relevant contexts. Nonapeptides are, therefore, highly likely to play an important and nuanced role in modulating reproductive success and mating tactics via structures associated with memory.

## A nonapeptidergic socio-spatial memory circuit

Based on the material discussed above, I propose that the influence of VP and OT in a putative “socio-spatial memory neural circuit” shapes reproductive decisions. In the remainder of this article, I attempt to outline this neural circuit in which the brain areas that contribute to social decision-making (and pairbonding in particular) interface with social and spatial memory processing to enable animals to successfully navigate and operate within a social context. Considering that successful mating tactics necessarily rely on an individual's ability to locate mates and competitors in space and are often related to (if not defined by) space use, it is probable that social and spatial memory have coevolved to—at least in part—serve the purpose of facilitating social behavior and mating success. The composition of the proposed network is based largely on neuroanatomical studies of connectivity between structures subserving social behavior and/or memory. Specifically, I refer to the extensive connectivity among the components of the limbic memory circuit, and their axonal connections with core areas within the pairbond neural circuit described throughout this review. In abstract terms, it is plausible that nonapeptide action in this memory circuit functions to integrate socio-spatial information to shape mating decisions in a context-dependent fashion. This context dependency is a notion supported by work demonstrating that social recognition varies based on the social environment in which it is tested (Zheng et al., 2013a).

This hypothesis predicts that neuromodulation by VP and OT in the memory circuit functions to evaluate the social landscape for potential mating and bonding opportunities. The degree to which these areas enable an animal to accurately account for the identity and location of conspecifics (mates and competitors) would be fed into the SDMN and specifically the pairbonding neural circuit. These behavioral networks could use that information to weight the probabilities that reproductive success can be maximized based on engaging in certain reproductive behaviors. Thus, communication between these nonapeptide sensitive circuits could shape reproductive tactics by biasing decision-making for remaining single or forming (faithful or unfaithful) bonds. The functional evidence discussed below is based on observations in prairie voles, which I use here as an example of how this might work.

**Figure 2:**
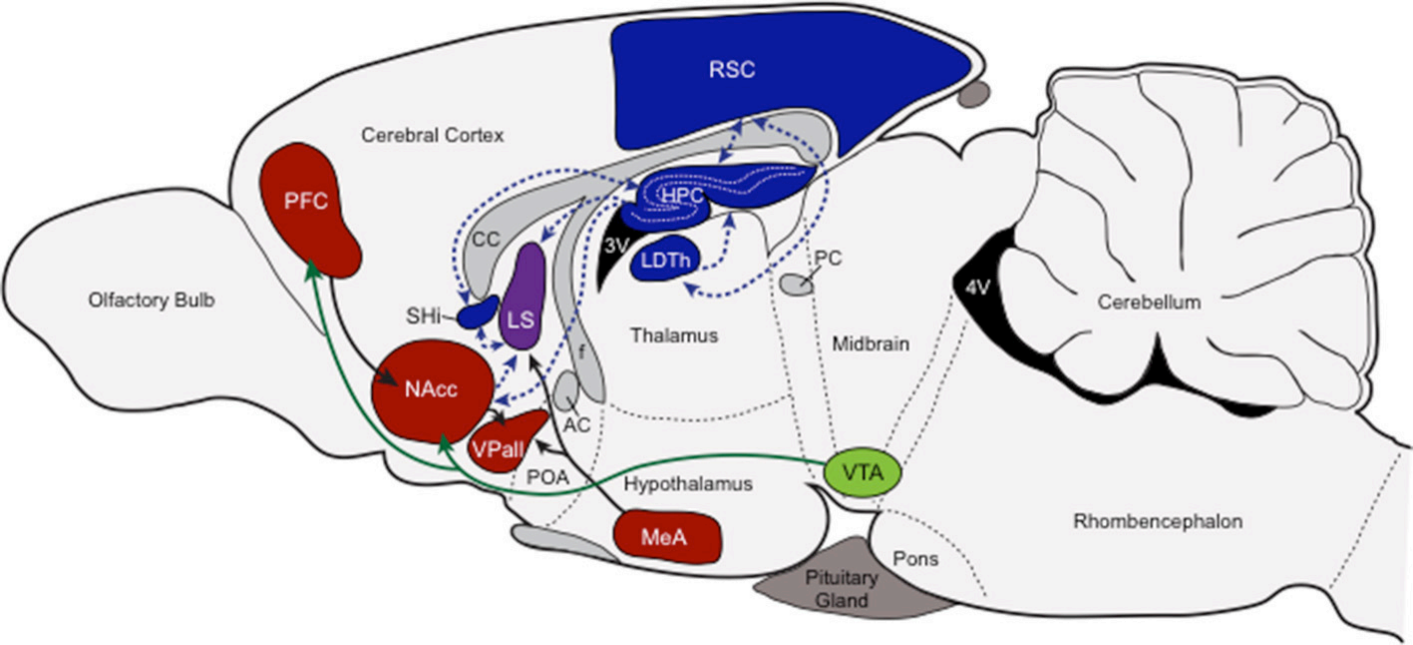
Putative socio-spatial memory neural circuit. This figure provides a cartoon schematic of the connections among the nonapeptide expressing memory areas, the connections among the pairbonding neural circuit, and the connections between these two circuits to showcase how socio-spatial memory might influence reproductive decision-making. Neural structures involved in processing socio-spatial memory (blue), and their projections (blue dashed arrows), include the retrosplenial cortex (RSC, V1aR) the laterodorsal thalamic nucleus (LDTh, V1aR), the hippocampus (HPC, OTR), and the septohippocampal nucleus (SHi, OTR). Neural structures involved in the pairbonding neural circuit (adapted from Young and Wang, 2004), and their projections, are also presented. Structures of this circuit that contain either V1aR or OTR (red) include the prefrontal cortex (PFC, OTR), nucleus accumbens (NAcc, OTR), ventral pallidum (VPall, V1aR), and medial amygdala (MeA, V1aR); the dopaminergic ventral tegmental area (VTA; green) is also included. The lateral septum (LS), pictured in purple, contains both OTR and V1aR and can be considered both a “memory” and “social behavior/pairbonding” node. Other abbreviations: CC, corpus callosum; AC, anterior commissure; POA, preoptic area; f, fornix; PC, posterior commissure; 3V and 4V, third and fourth ventricles.

At the center of this putative nonapeptide-governed socio-spatial memory circuit is the HPC, RSC, LDTh, SHi, and the LS (see Figure 2). With the exception of the LS, each of these areas demonstrates profound individual variation in either V1aR or OTR across individuals, indicating that variable sensitivity to the neuromodulatory influences of nonapeptides in these structures can account for individual variation in behavioral outcomes. Individuals also demonstrate the same clearly stereotyped patterns of nonapeptide receptors in the RSC, LDTh, HPC, and SHi, and these patterns predict reproductive success of those individuals based on their chosen mating tactic. Specifically, successfully breeding residents express the greatest densities of RSC and LDTh V1aR and HPC and SHi OTR, while successfully breeding wanderers express the least (Ophir et al., 2008b, 2012). Although neither V1aR nor OTR density shows this pattern in the LS, V1aR expression in the LS does show a non-significant trend that is consistent with the patterns seen in these four other structures (Ophir et al., 2008b). Moreover, OTR density in the HPC, SHi, and LS is significantly and positively correlated across these structures (Ophir et al., 2012), further supporting the idea that nonapeptide action coordinates the modulation of this network of memory processing brain structures.

Taken together, there is a strong precedent for the HPC, RSC, LDTh, SHi, and septum to either directly contribute to, or indirectly aid in, the processing of social and spatial memory. The connectivity and coordinated VP/OT sensitivity among these structures suggests an integrated network of memory-related structures. Functionally, this network could contribute to solving the cognitive demands of mating tactics within a social system. The main memory-processing components of this circuit (HPC, RSC, LDTh, and SHi) may make it possible to also process socially contextual information defined, in part, by the density and distribution of conspecifics in the surrounding environment. This premise is tentatively supported by the data discussed above.

The functional relationships and connectivity among these structures suggest that male prairie voles may rely on socio-spatial memory processing to shape the behavioral phenotype demonstrated by monogamous residents (including true residents and rovers) and non-monogamous wanderers. Within this framework, the LDTh and RSC influenced by VP, and the HPC and SHi influenced by OT may function to process context dependent learning and memory. But for this information to be useful in shaping reproductive behavioral outcomes, these “socio-spatial memory” structures would need to interface with the SDMN, and in this case specifically with the pairbonding neural circuit (Figure 2).

## Where memory and social behavior meet: connections to the pairbond neural circuit

I have suggested that the purported circuit detailed above may function to assess the socio-spatial context, enabling males to evaluate the probable reproductive value of forming bonds with females. The mechanisms for establishing and maintaining a pairbond have been relatively well characterized and are briefly summarized above (i.e., the pairbonding neural circuit, Young and Wang, 2004). In some instances the probability of forming bonds should be high. For example, males should be predisposed to form mating-induced bonds with females when such opportunities arise. This should be particularly true when pairing opportunities are promising because pairing appears to boost reproductive success (Ophir et al., 2008b; Okhovat et al., 2015; Blocker and Ophir, 2016). On the other hand, males should never forgo the opportunity to mate, even if it is unlikely to lead to a pairbond. In practice, if a male finds itself in a social context where several females are present but none is available for pairing, it would still greatly benefit from mating, but not pairing with those females. In fact, forming bonds with unavailable females would pose a great cost to males. In each of these cases, it would be important for the socio-spatial neural circuit to communicate with the pairbonding neural circuit and have the capacity to adjust the probability that a bond will form when mating occurs.

Where might the socio-spatial memory circuit interface with the pairbonding circuit? The lateral septum is one place where the two circuits converge. The LS is potentially unique in its role in relating learning and memory with social decision-making for several reasons. First and foremost, the LS can be considered a “memory” structure, a “social behavior” structure, or a “pairbonding” structure. Indeed, the LS, which is sensitive to both OT and VP, is important for many forms of social behavior (Goodson and Thompson, 2010), including social recognition (Ferguson et al., 2002; Gabor et al., 2012), and for establishing pairbonds (Liu et al., 2001). Interestingly, the action of VP in the LS, appears to be specific to learning and memory of social but not non-social information (Everts and Koolhaas, 1997). The LS's necessary and sufficient role in pairbond formation could be interpreted as enabling animals to make associations between the highly rewarding experiences from social affiliation and mating with the identity of a particular individual (Young et al., 2005). Therefore, in this and many other ways, the LS is most likely functioning as a general “association maker.” In the context of the proposed neural circuitry, the LS could aid in identifying the relative roles each conspecific might play in that individual's life (i.e., same-sex competitor, pair-mate, or non-mate female). Alternatively, the LS could promote or inhibit social grouping preferences, coloring the valences associated with learning the identities of neighbors and their relationships in space (Goodson and Wang, 2006; Goodson et al., 2009a,b; Kelly et al., 2011).

The nucleus accumbens is another particularly promising candidate area for integration of memory and social behavior. In particular, accumbal OTR densities might modulate hedonic interactions, biasing males to either form pairbonds or remain single. The NAcc is an integral component of the pairbonding neural circuit and has a well-established functional role in reward (Berridge and Robinson, 1998; Salgado and Kaplitt, 2015). Manipulations of oxytocin, dopamine, or mu-opioid receptors in this structure can alter the propensity to form bonds (Johnson and Young, 2015), and OTR density in the NAcc may modulate bonding by altering the intensity of reward (Liu and Wang, 2003; Aragona et al., 2006; Ross et al., 2009). Monogamous species of voles have higher densities of OTR in the NAcc than non-monogamous species (Insel and Shapiro, 1992; Insel et al., 1994), and OTR density in the NAcc is greater in paired resident prairie voles than the un-paired wandering males (Ophir et al., 2012). The NAcc is also the only pairbonding neural structure that differs between paired residents and single wanderers (Ophir et al., 2012). Furthermore, NAcc OTR is positively associated with OTR expression in several other important neural structures central to social decision-making including the prefrontal cortex and the amygdala (Ophir et al., 2012). Importantly, the NAcc receives strong projections from the HPC and LS, and it sends afferents to the LS and other limbic structures (Powell and Leman, 1976; Swanson and Cowan, 1977; Kelly and Domesick, 1982; Groenewegen and Russchen, 1984), suggesting that it is well positioned to serve as a relay center between the memory processing circuit outlined above and the social decision-making and pairbonding circuits (Figure 2).

Might the NAcc serve as a “tuning knob” (Young and Hammock, 2007) to bias males to bond or remain single? As just stated, the difference between adopting monogamous or non-monogamous tactics is related to OTR differences observed in the brain. In other words, OT may govern the behavioral differences in mating tactics via an OTR density-dependent neuromodulatory influence. With greater OTR density in the NAcc, resident males should be more sensitive to OT-modulated reward associated with mates. But, these data do not make it clear if the NAcc OTR phenotype preceded bonding in the field (i.e., a fixed phenotype that predicted the probability of bonding) or if it is dynamic and responsive to the social environment. Dynamic OTR in the NAcc could make it possible for animals to adjust their affiliative responses based on the context and, indeed, perception of accumbens-mediated reward can change based on the social context (Thiel et al., 2008). As it turns out, OTR in the NAcc is dynamic and epigenetically regulated, and this flexibility alters the likelihood that male and female prairie voles will form bonds (Wang et al., 2013; Duclot et al., 2016). Thus, accumbal OTR density could dynamically change based on the socio-spatial context, thereby altering the valence of the reward associated with mating. As a result, the reward associated with mating with a particular individual may only be sufficient to induce pairbonds when the social context is judged to be optimal or appropriate for forming bonds. Such changes in NAcc OTR could therefore impact normal functioning of the pairbonding neural circuit, enhancing or curtailing the probability of pairbond formation. If this is true, OTR in the NAcc could play a pivotal role as a bridge between socio-spatial neural structures that predict monogamous mating tactics, and neural structures that enable monogamous bonds to form. Further, OT action in the NAcc may broadly impact the SDMN, which could have a cascading effect on other aspects of sociality, thereby contributing to much larger behavioral consequences beyond bonding.

## Do resident and wanderer brains show distinct nonapeptide patterns?

It is clear that monogamous resident and non-monogamous wandering male prairie voles demonstrate distinct behavioral phenotypes, and that aspects of their brains differ (see above). To explore just how different these neural phenotypes are, I conducted hierarchical clustering analysis (JMP 12.0; SAS) of previously published nonapeptide receptor expression in pairbonding [VPall, NAcc, LS, MeA, and prefrontal cortex (PFC)] and memory (HPC, SHi, LDTh, RSC) areas (see Figure 2) taken from monogamous residents and non-monogamous wanderers living freely in outdoor semi-natural enclosures (for details see Ophir et al., 2008a,b, 2012). Hierarchical clustering groups data using an association matrix of pairwise *r*-values (for example, see Ophir et al., 2009). Thus, the correlations within each matrix provide a description of how well the relationships among variables relate to each other. It should be noted that cluster analyses like these make no *a priori* assumptions about grouping order or strength. Several interesting patterns are notable from this analysis, however I will focus on just two.

**Figure 3:**
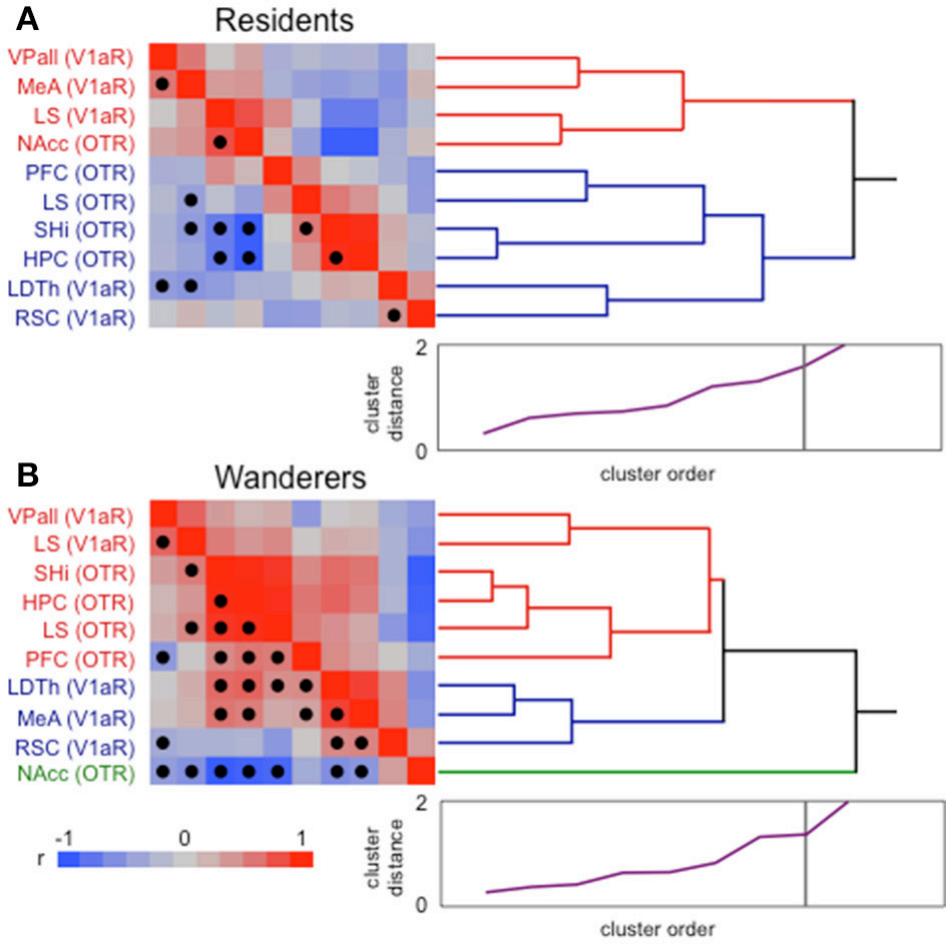
Hierarchical clustering analysis of resident and wandering male prairie voles. The clusters are composed of vasopressin receptor 1a (V1aR) and oxytocin receptor (OTR) expression in “memory” and “pairbonding” neural structures of paired residents **(A)** and single wanderers **(B)** living freely in semi-natural enclosures. Ward linkages were used in these analyses. Correlation matrices (on left) present strength (hue) and direction of correlations (red positive and blue negative; see legend at bottom) used to create the dendrograms (on right). False discovery rate-adjusted significant relationships are marked with a solid circle. For residents **(A)**, adjusted alpha = 0.0144, and significant p's ranged from 0.0067 to < 0.0001. For wanderers **(B)**, adjusted alpha = 0.0311, and significant p's ranged from 0.0302 to < 0.0001. Scree plots (bottom right of each panel, purple line) have a point for each cluster join. The ordinate (0–2) is the distance that was bridged to join the clusters at each step. Often, there is a natural break where the distance jumps suddenly. These breaks suggest natural cutting points to determine the number of clusters. The length of the branches in the dendrogram tree diagram is on a distance scale and shows the actual joining distance between each join-point. Thus, the longer the branch lines are, the larger the difference between samples having a common link. HPC, hippocampus; LDTh, laterodorsal thalamus; LS, lateral septum; MeA, medial amygdala; NAcc, nucleus accumbens; PFC, prefrontal cortex; RSC, retrosplenial cortex; SHi, septo-hippocampal nucleus; VPall, ventral pallidum.

The most striking pattern that these analyses revealed is that resident male brains show two branches of tight clustering; one comprised of most of the pairbonding-associated structures (VPall, MeA, NAcc), and the other containing all of the memory-associated structures (HPC, SHi, LDTh, RSC) (Figure 3A). The LS, which expresses both OTR and V1aR was split between these two clusters (LS V1aR in the “bonding branch”, and LS OTR in the “memory branch”), potentially reflecting its multifaceted role in bonding and memory. Although, OT action in the PFC, which clustered with memory structures, has been implicated in pairbonding (Young and Wang, 2004; Smeltzer et al., 2006), it is important for many forms of memory and primarily implicated in goal-directed behavior (Miller and Cohen, 2001).

In contrast to the clear pattern seen in residents, wanderers show a much greater degree of intermingling of OTR and V1aR expressing memory and bonding neural structures (Figure 3B). One interpretation of this pattern is that structures that contribute to these two different behaviors are non-distinct and show little cohesion, suggesting that the wanderer brain has little structure distinguishing between nonapeptide sensitive memory and bonding areas. Another interpretation of these results is that the nonapeptide-regulated structures that subserve bonding and memory are highly integrated. The latter interpretation is supported by the fact that, compared to the resident cluster, more of the correlations that were used to construct the wandering cluster were significant (following the false discovery rate correction for multiple comparisons). But how such integration across the two circuits operates, whether they work to improve or interfere with memory, and/or how that information is ultimately related to mating decisions remain interesting and unanswered questions.

A second noteworthy feature of these cluster analyses is the placement of the NAcc in the two clusters (Figure 3). In residents, it is closely associated with other structures that, like itself, are necessary and sufficient to induce pairbonds in prairie voles (see above). In wanderers, however, it was excluded from the branch containing all the other structures, effectively creating a single branch on its own. This is despite several significant (and negative) correlations with almost all the other structures that were fed into the analysis. These results are even more interesting considering that OTR density in the NAcc was significantly greater in residents than wanderers (Ophir et al., 2012). Perhaps the “isolation” of NAcc OTR from the other structures in the wanderer brain is another reflection of its potentially pivotal role as a node enabling/preventing communication between structures associated with reproductive decision-making and socio-spatial memory.

Cluster analyses such as these are useful to get a general sense of potential relationships across the brain. In this case, a main point is that resident and wandering brains demonstrate very distinct patterns of V1aR and OTR expression within these two circuits. Unfortunately, it is difficult to make specific functional conclusions from descriptive analyses like these. Nevertheless, these data clearly demonstrate that individuals that have adopted two distinct alternative reproductive tactics also demonstrate different broad-scale neural phenotypes. The different patterns of nonapeptide receptor associations have the potential to shape memory processing and pairbonding in very different ways by acting on the coordination of networks of nuclei that are potentially important for evaluating the social landscape and shaping mating tactics.

## Concluding remarks

The ability to navigate space and relate that ability to social interactions is something that has been relatively unappreciated in discussions of mating system. I have attempted to make the case that these behaviors are integral to mating systems and in particular for successful monogamy. I have provided evidence supporting the hypothesis that neural mechanisms involved in socio-spatial memory shape the mating decisions resulting in differential mating tactics, and that these processes are functionally modulated by nonapeptides (VP and OT). Such data have led to the hypothesis that a putative “socio-spatial memory neural circuit” informs reproductive decisions. Presumably, this putative network enables prairie voles to assess the social landscape and bias their decision-making for remaining single or forming (faithful or unfaithful) bonds to maximize their probability of reproductive success in nature. Such a decision-making process largely accounts for the form of mating system prairie voles demonstrate (i.e., social monogamy with multiple alternative reproductive tactics). Importantly, despite species differences that are sure to exist, the larger function of this circuit—assessing the social and spatial landscape to inform reproductive decision-making—is likely to be a general feature of brains in many species. Therefore, this putative circuit need not be limited to explaining the interface between memory and reproductive decisions in prairie voles. Indeed it is likely to extend beyond addressing reproductive decisions related specifically to monogamy.

The extensive connectivity among the memory-related brain structures, and their axonal connections with core areas within the pairbonding neural circuit appears to form a larger network of structures, distinct in their functions but bound by their shared sensitivity to nonapeptides. This provides a foundation on which this network has the potential to subserve the larger (and emergent) behavioral function of integrating socio-spatial information to shape mating decisions in a context-dependent fashion. I have argued that these “memory” structures are likely to work with the SDMN via the LS and NAcc to enable the evaluation of the social landscape to weight reproductive decisions that determine individual mating tactics and ultimately mating systems. It is clear that this hypothesis will require sufficient testing, but I have aimed to provide a framework from which novel hypothesis and new predictions can be generated. Ultimately, I hope that this article broadens the discussion of social and spatial memory, mating systems and social behavior, and inspires crosstalk between these fascinating and inextricably linked areas of research.

## Author contributions

The author confirms being the sole contributor of this work and approved it for publication.

### Conflict of interest statement

The author declares that the research was conducted in the absence of any commercial or financial relationships that could be construed as a potential conflict of interest.

## Abbreviations

AC: Anterior Commissure
AT: Anterior Thalamus
BST: Bed Nucleus of the Stria Terminalis
CeA: Central Amygdala
CC: Corpus Callosum
DG: Dentate Gyrus
EC: Entorhinal Cortex
f: Fornix
HPC: Hippocampus
LS: Lateral Septum
LDTh, Laterodorsal Thalamus, MB: Mammillary Bodies
MeA: Medial Amygdala
MS: Medial Septum
NAcc: Nucleus Accumbens
PAR: Parietal Cortex
PAG: Periaqueductal Grey
PFC: Prefrontal Cortex
POA: Preoptic Area
PC: Posterior Commissure
RSC: Retrosplenial Cortex
SHi: Septohippocampal Nucleus
Sub: Subiculum
VPall: Ventral Pallidum
VTA: Ventral Tegmental Area
3V and 4V, Ventricles: third and fourth
